# Plant trait diversity buffers soil moisture dynamics on coastal dikes during drought periods

**DOI:** 10.1371/journal.pone.0345552

**Published:** 2026-03-26

**Authors:** Jan-Michael Schönebeck, Dorothea Bunzel, Maike Paul, Torsten Schlurmann

**Affiliations:** 1 Ludwig Franzius Institute of Hydraulic, Estuarine and Coastal Engineering, Faculty of Civil Engineering and Geodetic Sciences, Leibniz University Hannover, Hannover, Lower Saxony, Germany; 2 Institute of Earth Sciences, Faculty of Chemistry and Earth Sciences, Ruprecht Karl University of Heidelberg, Heidelberg, Baden-Württemberg, Germany; University of Deusto: Universidad de Deusto, SPAIN

## Abstract

Soil moisture is considered a key component for the structural integrity of engineered ecosystems, such as sea dikes. Although plants are important determinants of physical soil properties in dike greening, research lacks on the extent to which greater biodiversity can mitigate soil moisture loss during extreme weather events. This provided the motivation to investigate the influence of two plant communities of different species composition – namely, an herb-dominated vegetation area (‘Mix-Herb’) compared to a grass-dominated area (‘Mix-Grass’) – on soil physical conditions over the course of one year on a summer dike in northern Germany. Vegetation mapping, high-resolution measurements of soil temperature and moisture, and comprehensive precipitation data provided the framework for the investigations. It was found that species diversity (Shannon Index) declined over time from 2.7 to 2.3 for ‘Mix-Herb’ and from 2.2 to 2.0 for ‘Mix-Grass’. In-situ measurements of soil physical conditions revealed that the ‘Mix-Herb’ plant community moderated diurnal soil temperature variations more effectively than ‘Mix-Grass’. During a drought in June 2023, the ‘Mix-Herb’ vegetation area was also considerably less affected by soil heating and moisture deficit. However, after mowing, the thermal buffer effect reversed and greater diurnal temperature variations occurred in the soils of the herbaceous vegetation. During a second drought in September 2023, the’Mix-Grass‘soils exhibited higher moisture loss rates after mowing. These findings highlight the importance of the functional composition of plant communities and management practices such as mowing schedules, tailored spatially and temporally to ecological and climatic conditions, for regulating the soil microclimate on dike systems, with potential implications for dike’s resistance under climatic extremes.

## 1. Introduction

In recent decades, the global environmental discourse has been increasingly dominated by the subject of climate change [1]. The alarming rise in global land and ocean surface temperatures and the increasing frequency of extreme weather events such as heavy rainfall, flooding, droughts and heatwaves [2] have attracted the attention of politicians and scientists, but also that of the general public and thus brought climate policy into the public debate (e.g., [3–5]). However, this great interest in climate change has overshadowed another central issue of global environmental policy, namely the loss of biological diversity (or biodiversity), a second planetary boundary that will lead to a change in the Earth system in the long term [6,7].

Biodiversity loss, characterized primarily by the rapid decline in species abundance and species richness, is due to various cumulative pressures that include both climatic drivers, such as the impacts of climate warming, extreme weather events, increased atmospheric CO_2_ concentrations, oceanic heat uptake, weakened thermohaline circulation as well as ocean deoxygenation and acidification (e.g., [8–11]) and non-climatic drivers, such as the fragmentation and degradation of habitats, overexploitation of resources, environmental pollution or the spread of alien species (e.g., [12–15]). Often these drivers interact and reinforce each other, complicating the efforts to overcome the underlying main causes [10,16].

Although some studies indicate that individual plant species are capable of transgenerational adaptation to changing environmental conditions [17], such plant resilience is not universal. Consequently, the loss of biodiversity jeopardizes the stability of an ecosystem and can ultimately lead to its collapse [18,19], resulting in the loss of critical ecosystem services that human society rely on [20]. In particular the variety of biological traits of organisms in an ecosystem is closely linked to ecological processes that enable key ecosystem services [21,22]. For example, the loss of functional characteristic plant species on coastal margins can lead to the loss of regulating ecosystem services, such as coastal protection services, generally provided by halophytic plant communities (e.g., salt marshes; [23,24]). If a shoreline-stabilizing, flood-reducing and wave-attenuating plant community is degraded due to the decline in biodiversity, this impairs its functional diversity and thus the regulating services of the system (e.g., [22,25,26]). In shallow coastal areas in particular, which are exposed to greater wave loading, such functional losses can lead to increased coastal erosion as well as higher wave run-up and wave overtopping on conventional engineered coastal protection structures such as dikes, which in turn entails high coastal damages and economic costs [23,27]. Biodiversity loss is therefore a multidimensional challenge that poses a major threat to the planet’s ecological balance [7,28,29], but it also has consequences at the socio-economic level [30,31]. Despite its critical impact, however, biodiversity loss has not yet received the same urgency and level of action as climate change.

The question therefore arises how plant biodiversity in coastal areas can be specifically promoted in order to strengthen the ecosystem’s resistance and adaptability to the effects of climate change and thus its functionality with regard to its coastal protection services. However, conventional engineered coastal protection structures are also getting under increased threat from the effects of climate change, including accelerated SLR, elevated storm surge and wave run-up levels, as well as increased wave action [32]. This also underscores the importance of further focusing on the vegetation directly on dikes to better understand their ecological functionality and their potential in terms of resistance and adaptability to climate-related pressures. Therefore, new guiding principles for coastal management and future-proof coastal protection solutions are needed that complement rather than replace hard engineering solutions to achieve the highest level of societal acceptance, while ensuring additional benefits for climate protection and nature conservation through the promotion of biodiversity [33]. In this context, ecosystem-based coastal defences, a combination of natural ecosystem elements and hard engineering structures, represent a future-oriented approach that simultaneously offers a shift towards greener design [34], climate change mitigation [35] and cost-effective support for traditional engineering solutions, such as sea dikes [36–38]. A promising and novel implementation of ecosystem-based coastal defences appears to be the targeted planting of sea dikes with more biodiverse plant communities.

Conventionally, a dense grass sward is established on sea dikes, supposed to provide protection against wave impact in case of a storm surge and soil drying during droughts and thus against the formation of desiccation cracks, but takes little account of biodiversity or allows for variations in ecosystem functions [33,39]. The main body of sea dikes is usually designed with a sandy dike core and a subsoil made up of cohesive clay soil [40]. Since clay soil consists primarily of fine-grained sediments, its main function is to build a first erosion-resistant barrier and to protect the sandy dike core against wave erosion [41]. Its properties make clay soil particularly suitable for sealing dikes, but also tend to form desiccation cracks when exposed to drought, enabling water to infiltrate the dike’s interior and endanger its coastal protection function [40,42,43]. The level of soil moisture required to prevent desiccation cracks is determined by a host of factors beyond weather conditions, including soil type, dike maintenance, vegetation structure and plant species diversity [44], which also influence each other. For example, the type of maintenance can lead to a decline of plant biodiversity and soil biota at the local level, especially if key factors that control soil processes, such as soil temperature and moisture, are considerably altered as a result [45,46]. However, little is known about how species-rich plant communities affect the stability of dikes and what influence different plant traits have on soil temperature and moisture. In particular, there is a lack of long-term *in-situ* measurements of soil temperature and moisture on dikes as a function of different plant compositions and their responses to changing weather conditions toward more extreme events such as droughts.

Here we present the first attempt to observe and describe potential relationships between plant communities of different species richness and trait diversity and soil temperature and moisture as well as the prevailing climatic (such as droughts) and non-climatic influences (such as dike maintenance) on a dike. The overarching objective is to identify the key drivers that contribute to the resistance of vegetated dikes in order to better compensate for regional climate impacts and improve ecosystem services at all levels through increased biodiversity. To this end, a summer dike on the North Sea coast of Lower Saxony (Wesermarsch, Germany) was sown with two seed mixtures of different numbers and weight percentages of plant species (mainly belonging to the Asteraceae, Fabaceae, Plantaginaceae and Poaceae) and the established plant communities and diversities were documented and quantified after two years. During the time period from a closed grass sward to the final vegetation evaluation, a one-year and continuous *in-situ* soil monitoring was carried out in order to record changes in soil temperature and moisture and finally to be able to link the collected vegetation and soil data with regional meteorological observation data (such as droughts). On this basis, this study addresses the following questions: (1) How does the composition of seed mixtures of different diversity influence the development of plant communities and thus the functionality of the system? (2) To what extent does dike vegetation with a higher biodiversity have a positive effect on soil temperature and moisture under extreme meteorological conditions? (3) What influence does maintenance (mowing) have on the soil temperature and moisture?

## 2. Materials and methods

### 2.1 Regional setting and experimental setup

**Fig 1:**
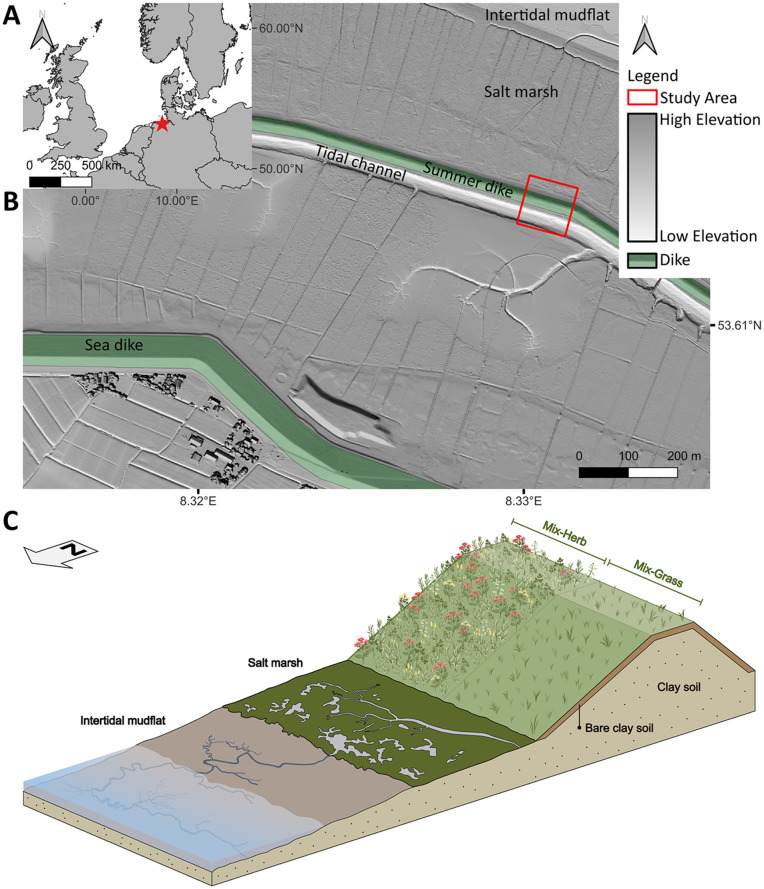
Location of the study area and the experimental setup of the two planted dike sections. (A) Overview of the study area at the southeastern North Sea coast, Germany, on the Butjadingen Peninsula, Wesermarsch (red star). (B) The summer dike in front of the main sea dike (shaded in green), on which the two dike sections were investigated (red square). (C) Simplified cross-section of the summer dike according to the experimental setup with a 20 cm thick layer of new bare clay soil and the two established plant communities ‘Mix-Herb’ (herb-dominated) and ’Mix-Grass’ (grass-dominated); not to scale. The maps (A-B) were created using Natural Earth (CC0, public domain) and DOM1 data republished from OpenGeoData NI under a CC BY license, with permission from Landesamt für Geoinformation und Landesvermessung Niedersachsen (LGLN), original copyright 2020.

The summer dike system located at the southeastern North Sea coast of Germany, between the Jade Bight and the Weser estuary on the Butjadingen Peninsula, Wesermarsch (53°36'44.1''N, 008°19'50.6''E; Fig 1A), offers ideal conditions for achieving the aforementioned research questions and objective, as the summer dike is no longer a legally designated part of the primary coastal protection system and therefore the hinterland is not endangered during the experimental phase. The investigated summer dike has a dike crest of around 3.6 m above mean high water (MHW) and is therefore considerably higher than conventional summer dikes along the North Sea coast that are commonly 2 m above MHW [41]. This means that the summer dike studied is only flooded during very severe storm surges [47]. Although the height of the summer dike crest is lower than that of the main sea dike (>8 m MHW; [41]), but with its broad offshore salt marsh, it is therefore representative and typical for dike systems on the German North Sea coast (Fig 1B). Despite the recommendations for the design and construction of coastal protection structures [41], the dike studied only consists of clay soil and does not have a sand core, which, however, does not have any negative impact on the following investigations.

A northeast facing 24 m long area of the summer dike was divided into two equally sized sections on which two different seed mixtures were sown on September 24, 2021 comprising varying proportions of different plant seed species mainly belonging to the Asteraceae, Fabaceae, Plantaginaceae and Poaceae (Fig 1C, S1 Table). The seed mixtures were compiled on the basis of vegetation mapping of existing dikes and coastal systems [48]. The functional traits (e.g., plant height, growth habit, rooting depth) of the recorded plant species were integrated into a database by the authors, enabling plant species with a high degree of similarity to established dike plant species to be identified and combined using principal component analysis. In the present study, the seed mixtures that had proven to be the most suitable for greener dike design in previous experiments on test dikes [49] were then selected. Both seed mixtures thus contained a substantially higher number of species than is mandatory for conventional dike seed mixtures consisting mainly of rhizomatous grasses (Poaceae), i.e., *Lolium perenne* (30 wt.%), *Poa pratensis* (30 wt.%), *Festuca rubra* ssp. *trichophylla* (25 wt.%) and *Festuca rubra* ssp. *rubra* (15 wt.%). Asteraceae (e.g., *Achillea millefolium*) can be added with a maximum of 3 wt.% at the expense of *L. perenne* [41].

Prior to sowing, the existing vegetated topsoil of both dike sections studied was removed to a depth of 20 cm and then filled with new bare clay soil to avoid the presence of old root residues or seeds (Fig 1C). The filled bare clay soil was first compacted and then the upper few centimeters were loosened with a harrow. The whole area was then fertilized with 53 g m^-^² of an organic-mineral fertilizer (containing 9 wt.% total N (incl. 3 wt.% urea-N) from animal by-products, KCl, (NH₄)₂SO₄, 3 wt.% MgO and 41 wt.% organic matter from plant residues). One dike section was then sown with an herb-dominated seed mixture, containing 16 species, 10,590 seeds m^-^² with 2% grass and 98% herb species (hereafter referred to as ‘Mix-Herb’ seed mixture; see S1 Table). The second dike section was sown with a grass-dominated seed mixture, containing 10 species, 10,030 seeds m^-^² with 80% grass and 20% herb species (hereafter referred to as ‘Mix-Grass’ seed mixture; see S1 Table). For the sake of simplicity, all sown species belonging to the Asteraceae, Caprifoliaceae, Fabaceae, Lamiaceae, Malvaceae and Plantaginaceae were jointly referred to as ‘herbs’. The number of seeds per square meter was reduced, contrary to the EAK [41], to minimize competitive pressure between the plant species as far as possible [48]. The respective seed mixtures were spread by hand on the two sections of the summer dike to avoid separation of the seeds due to differences in density and then raked under. Both sections of the dike were mowed once a year, in August 2022 and August 2023, using a mulching mower. The cut plant residues were left on the respective dike sections as mulch layer. The choice and sowing of seed mixtures, the redesign of the dike and the installation of the soil sensors was coordinated with and granted by the Lower Saxon Wadden Sea National Park Administration (Nationalpark Niedersächsisches Wattenmeer; permit number 01.2-22242-1-1.0 (8–9)/ 2021). Damage to the surrounding vegetation was kept to a minimum. The entire summer dike is generally used as agricultural land from May to October and is mowed once a year in late summer. The annual mowing and its timing were adopted and continued as part of the study. The area is freely accessible all year round.

### 2.2 Soil analyses

To understand how plant biodiversity influences soil conditions, high-resolution temporal soil analyses were carried out on both dike sections, with a particular focus on the variability of soil temperature and moisture and how different vegetation types affect these parameters, which are critical for dike stability. Once the newly sown seed mixtures had successfully established after one year and a uniform vegetation coverage had developed on both dike sections, a total of six soil sensors (TEROS-12 with ZL6 Funk Data Logger, METER Group AG) were installed in November 2022. Three sensors were installed per vegetation area of the ‘Mix-Herb’ and ‘Mix-Grass’ seed mixtures at soil depths of 4 cm, 14 cm and 24 cm to monitor both the soil temperature and moisture at the surface and in the root zone every 10 minutes. For reasons of simplicity and clarity, the 10-minute interval values were calculated to hourly average values in the subsequent data processing of soil temperature and moisture, which are used from here on. The soil sensors remained in the soil for one year, until November 2023, so that a continuous recording of the changes in soil temperature and moisture could be measured. All sensors were installed on the southern, inland facing side of the dike, approximately 1 m in elevation below the dike crest, where greater differences in diurnal and seasonal temperature and moisture variations in the soil are to be expected due to solar radiation. Since both the ‘Mix-Herb’ and the ‘Mix-Grass’ seed mixtures have different compositions of grasses and herbs (in terms of species richness and weight percentage, where the latter can be considered as the initial species abundance), were sown in the same new bare clay soils and developed under the same environmental and climatic conditions, the soil sensors allow long-term tracking of temporal changes in soil temperature and moisture as a function of the prevailing plant community.

Statistical analyses were carried out to investigate the differences between the recorded soil temperature and moisture values of the two dike sections. Since there is no normal distribution of the soil parameters (according to the Shapiro-Wilk test), the Mann-Whitney U test was used, which is less sensitive to outliers and compares the distributions of two similar shaped groups without assuming normality [50] making it suitable for environmental data. To prevent potential artificial significance resulting from a large sample size, the datasets were reduced by calculating the daily average soil temperature and moisture as well as the diurnal variations in soil temperature and moisture [51,52]. Based on the results of the Mann-Whitney U test, non-parametric effect sizes (Vargha-Delaney A) were calculated to evaluate the practical relevance of statistically significant results [52,53]. To account for potential effects of mowing on soil temperature and moisture, the dataset was divided into pre- and post-mowing measurements, and statistical tests were performed for each subset. All statistical analyses were performed using Python’s SciPy library version 1.8.1 in Spyder 4.2.5.

### 2.3 Vegetation analyses

In order to obtain detailed information on the two established plant communities, taxonomic species identification and relative plant species abundance was carried out by an external environmental office on both dike sections in June 21, 2023, i.e., nearly two years after sowing. In order to exclude the influence of salt water introduced by storm surges on the plant composition, mapping was carried out above a height of 1.8 m MHW and on both, the seaward and the landside slope of the dike, covering an area of 180 m² per vegetation area.

Species diversity (Shannon Diversity Index H[S]) was determined to obtain a quantitative estimate of the biological variability of both the sown seed mixtures and the subsequent established plant communities. The Shannon Index is defined by: $H(S)=(-\text{1})\sum_{i=\text{1}}^{S}p_{i}\;\times ln(p_{i})$, with *S* being the species richness and *p* the relative abundance of the *i*th species [54]. The Shannon Index was calculated on the assumption that the estimate of the percentage area coverage of each species acquired during the mapping represents its relative abundance in the section of dike studied. When calculating the Shannon Index for the established plant community on the ‘Mix-Grass’ area, however, it should be noted that the initially sown grass subspecies were not considered, as taxonomic identification at subspecies level was not possible in the field and thus a representative comparison between the diversity at the time of sowing and the mapping would not have been possible. Accordingly, the subspecies were summarized at the species level for the Shannon Index calculation.

Additionally, β-diversity was calculated using the Jaccard similarity index (J) to quantify the degree of species overlap between two sites or between two points in time [55]. The Jaccard index is a robust measure for comparing species richness between communities and is particularly suited for binary data (presence/absence). The Jaccard index is defined as the ratio of the number of shared species to the total number of unique species found across both sites. The formula is: $J=\;\frac{C}{A+B-C}$ where A is the number of species at the first site, B is the number of species at the second site and C is the number of species shared between both sites [55]. As with the calculation of the Shannon Index, the subspecies were summarized at species level for the calculation of the Jaccard index. The Jaccard index was calculated (i) at the time of sowing, based on the number of species of the ‘Mix-Herb’ and ‘Mix-Grass’ seed mixtures, and (ii) at the time of vegetation mapping. In addition, the Jaccard index was calculated (iii) within the ‘Mix-Herb’ and ‘Mix-Grass’ vegetation areas to assess temporal changes in species composition from sowing to mapping.

### 2.4 Meteorological data

Droughts were considered to be most critical for the resistance of plant and soils and thus for dike stability. Therefore, precipitation data provided by the German Meteorological Service (Deutscher Wetterdienst, DWD) were used for the weather station ‘Burhave’ (ID 827; 53°34'57.1''N, 8°22'07.8''E, station height is 2 m above mean sea level) that is located at the coast and at a distance of 4.1 km southeast of the study area. The data were provided in a resolution of daily recordings.

The standard precipitation index (SPI) [56] was used to identify defined drought periods on the basis of daily precipitation rates during the period from November 2022 to November 2023 to coincide with the period of soil temperature and moisture measurements. For the SPI calculations, the Python-based code developed by Wang et al. (2022, https://github.com/Wangqianfeng23/DailySPI) [57] was applied and a daily SPI with a rolling average of 30 days was calculated on the basis of a reference period from 1991 to 2020. The SPI ranges from negative to positive values, with values of −1.0 to −1.5 indicating moderate droughts (probability of occurrence is 9.2%), values of −1.5 to −2.0 indicating severe droughts (probability of occurrence is 4.4%) and values of −2.0 or lower indicating extreme droughts (probability of occurrence is 2.3%; [56]).

## 3. Results

### 3.1 Soil physical dynamics

**Fig 2:**
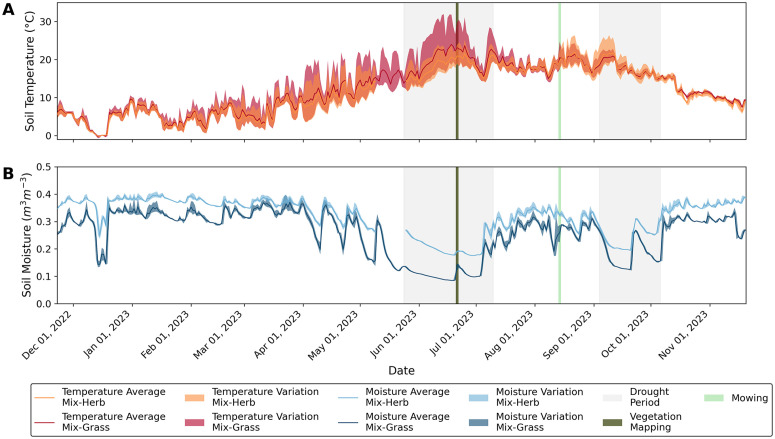
High temporal resolution soil temperature and moisture data reveal vegetation-depending differences. Daily minimum and maximum values of soil temperature (A, orange and red bands) and soil moisture (B, cyan and blue bands) with darker, bold lines for the corresponding daily averages of both parameters during the measurement period from November 22, 2022 to November 22, 2023. The soil temperature and moisture values are given for a depth of 4 cm and for dike sections ‘Mix-Herb’ and ‘Mix-Grass’. Key events are the two drought periods (gray bands), vegetation mapping (olive green band) and mowing (light green band). Data gap between May 9 and May 25, 2023, on the ‘Mix-Herb’ dike section was due to temporary sensor breakdowns.

The daily average soil temperatures calculated for the two dike sections showed no significant difference between the ‘Mix-Herb’ and ’Mix-Grass’ vegetation areas before (p = 0.26) or after mowing (p = 0.78), based on the Mann-Whitney U test (Table 1). In contrast, diurnal soil temperature variations before and after mowing were significant (p < 0.01), with the effect size being small before mowing and moderate after mowing [53]. There were also significant differences (p < 0.01) in average daily soil moisture between the ’Mix-Herb’ and ’Mix-Grass’, vegetation areas both before and after mowing, having a large effect size [53]. In contrast, the diurnal soil moisture variations were either not significant or only had a small effect (Table 1). The greatest changes, both in soil temperature and moisture, took place in the uppermost soil layers, at 4 cm depths. Nevertheless, the same temporal pattern of rising or falling soil temperatures and soil moisture was evident within all soil layers, although less intensively and slightly delayed with increasing depth (Fig 2, S1 Fig). As the highest diurnal differences in soil temperature and moisture occurred in the uppermost soil layers and the risk of desiccation cracks forming is greatest here, only the measured values from the sensors at a depth of 4 cm are discussed below.

**Table 1:** Statistical results based on the Mann-Whitney U test for soil temperature and moisture.

Variable	Periode	N	U	p	A
Average Soil Temperature	before mowing	500	33063	0.26	0.53
Average Soil Temperature	after mowing	204	5318	0.78	0.51
Soil Temperature Variation	before mowing	500	38838	<0.01	0.62
Soil Temperature Variation	after mowing	204	3166	<0.01	0.30
Average Soil Moisture	before mowing	500	15599	<0.01	0.25
Average Soil Moisture	after mowing	204	2607	<0.01	0.25
Soil Moisture Variation	before mowing	500	38307	<0.01	0.61
Soil Moisture Variation	after mowing	204	4991	0.62	0.48

N, total sample size; U, Mann-Whitney U-statistic; p, p-value based on Mann-Whitney U test; A, Vargha-Delaney A effect size.

Considering the entire measurement period from November 2022 to November 2023, the lowest soil temperatures for both dike sections were reached in mid-December 2022 with an hourly average of −0.3 °C (‘Mix-Herb’) and −0.7 °C (‘Mix-Grass’) at a shallow soil depth of 4 cm (Fig 2). In contrast, the highest soil temperatures were reached in mid-June 2023 with 31.9 °C for ‘Mix-Grass’ and at the beginning of September 2023 with 26.5 °C for ‘Mix-Herb’. When comparing the seasonal soil temperatures, it is noticeable that the soil temperature values in the winter months were almost the same on both dike sections, while the soil temperatures in summer differed greatly depending on the plant community prevailing on the respective dike section (Fig 2). In summer 2023, temperature differences of up to 8.5 °C were measured between the two sown areas, with the ‘Mix-Herb’ area showing lower average soil temperatures than the ‘Mix-Grass’ area. In addition, the surface soils of the dike section with the ‘Mix-Grass’ seed mixture showed greater diurnal temperature variations in June 2023, with average minimum temperatures of 17.0 °C (nighttime) and average maximum temperatures of 27.5 °C (daytime), than the dike section with the ‘Mix-Herb’ seed mixture, where average minimum temperatures of 16.4 °C and average maximum temperatures of 21.9 °C were documented at the same time. The measurements also showed that the average temperature in the soil of the herb-rich plant community (‘Mix-Herb’) during the first phase of the recorded drought period (May 24, 2023 to June 6, 2023 [see Chapter 3.3]) was on average 1.2 °C lower than in the soil of the adjacent vegetation area, which was dominated by grass species (‘Mix-Grass’). Most strikingly, the diurnal variations between minimum and maximum soil temperature were 66% higher in the grass-dominated plant community (8.6 °C on average, ‘Mix-Grass’) as in the herb-dominated community (only 5.7 °C on average, ’Mix-Herb’) in the same period of time. After mowing on both dike sections in August 2023, however, this pattern was reversed, so that the diurnal temperature variations were now higher in the soil of the herb-dominated vegetation area.

Overall, the soil moisture values of the two dike sections investigated showed the generally expected annual trend. That is, high soil moisture in spring, fall and winter, with maximum values of 0.410 m^3^ m^-3^ for ‘Mix-Herb’ and 0.398 m^3^ m^-3^ for ‘Mix-Grass’ at a depth of 4 cm and minimum values in summer of 0.175 m^3^ m^-3^ for ‘Mix-Herb’ and 0.084 m^3^ m^-3^ for ‘Mix-Grass’. Comparing the annual soil moisture values between the two dike sections with the different plant communities, it is noticeable that the soil moisture of the ‘Mix-Herb’ area was on average about 0.054 m^3^ m^-3^ ± 0.018 (75% percentile) higher than that on the dike section with the ‘Mix-Grass’ seed mixture (Fig 2). During the first drought period from mid-May to mid-July 2023, the soil moisture on both dike sections dropped to their minimum values. Due to the higher initial value, the vegetation area of the ‘Mix-Herb’ seed mixture showed a higher rate of decrease in soil moisture compared to the vegetation area of ‘Mix-Grass’. The soil moisture loss rates were 0.004 m^3^ m^-3^ d^-1^ for the ‘Mix-Herb’ area and 0.003 m^3^ m^-3^ d^-1^ for the ‘Mix-Grass’ area. During the second drought period from September 4 to September 11, 2023, the soil moisture of the vegetation areas dropped to 0,205 m^3^ m^-3^ (‘Mix-Herb’) and 0,142 m^3^ m^-3^ (‘Mix-Grass’). Both before and after mowing in August 2023, the soil moisture content in the ‘Mix-Herb’ area remained higher than in the ‘Mix-Grass’ area (Fig 2).

### 3.2 Vegetation patterns and plant biodiversity

At the time of vegetation mapping in Butjadingen in June 21, 2023, the ‘Mix-Herb’ area showed a vegetation coverage of 96% at a vegetation height of 50–150 cm, while the ‘Mix-Grass’ area showed a vegetation coverage of 98% at a vegetation height of 60 cm. The overall relative species richness derived by in-field taxonomical identification and mapping showed that both novel and biodiverse seed mixtures sown in Butjadingen in September 2021 developed well and still largely exhibited their originally sown species composition in June 2023 (S1 Table). However, the plant mapping also revealed that a few of the originally sown species had disappeared (e.g., *Trifolium fragiferum*), but that many new plant taxa had been added naturally, e.g., from the Amaranthaceae, Apiaceae, Plumbaginaceae, Ranunculaceae or Rosaceae, but also many new species belonging to the Poaceae (e.g., *Dactylis glomerata*, *Elymus* spp., *Festuca* spp., *Holcus lanatus*, *Poa* spp.). For the sake of simplicity, the newly introduced taxa (unless they were Poaceae) are summarized as ‘herbs’ from now on, while the new Poaceae will be referred to as ‘grasses’. Remarkable was the occurrence of *Pastinaca sativa* on the ‘Mix-Herb’ vegetation area, which is a typical indicator species for nutrient-rich alkaline meadows [58].

In June 2023, a total of 27 taxa were identified to species level on the dike section with the ‘Mix-Herb’ seed mixture, with the majority belonging to the group of herbs (15 different species) and the group of grasses being rather subordinate in comparison (12 different species). Diversity had decreased from the time of sowing (H(S) = 2.7) to the time of mapping (H(S) = 2.3), i.e., within two years. On the dike section with the ‘Mix-Grass’ seed mixture, a total of 18 taxa were identified at species level, whereby the species richness of the herbs was again higher (10 different species) than the group of grasses (8 identified species). Here, the Shannon Index showed only a smaller decrease in diversity from 2.2 (at time of sowing) to 2.0 (at time of mapping). Beyond that, both dike sections in June 2023 also showed great differences in terms of their species composition and their relative abundance: While the seeding area with the ‘Mix-Herb’ mixture was most dominated by Asteraceae (23% *Leucanthemum vulgare* and 15% *Cichorium intybus*) and Poaceae (23% *Arrhenatherum elatius*), Fabaceae (23% *Lotus corniculatus*), Poaceae (18% *Festuca pratensis* and 13% *Festuca rubra*), Asteraceae (14% *A. millefolium*) and Plantaginaceae (15% *Plantago lanceolata*) were the most abundant taxa on the dike section with the ‘Mix-Grass’ mixture.

The seed mixtures for ‘Mix-Herb’ and ‘Mix-Grass’ contained a total of 26 species, 5 of which were present in both seed mixtures (see S1 Table). This resulted in a Jaccard index (J) of approximately 0.24, which means that 24% of the species were found in both seed mixtures. At the time of vegetation mapping, a total of 35 different species were found, 10 of which were present on both vegetation areas (J = 0.29). Of the total of 16 species sown in the ‘Mix-Herb’ vegetation area, 13 species were still present at the time of mapping. In addition, 17 species had self-seeded there over time (J = 0.43). This means that at the time of mapping, 43% of the ‘Mix-Herb’ species still corresponded to the species composition that was present at the time of sowing. Of the 10 species sown in the ‘Mix-Grass’ vegetation area, 9 species were found during mapping, and 10 others had self-seeded (J = 0.47). According to the Jaccard index, this means that at the time of mapping, 47% of the species in the ‘Mix-Grass’ vegetation area corresponded to those that had been sown (S1 Table).

Of the 3 additional herb species and 11 additional grass species that colonized the ‘Mix-Herb’ vegetation area between sowing and vegetation mapping, 5 grass species were also included in the ‘Mix-Grass’ seed mixture. On the ‘Mix-Grass’ vegetation area, 6 additional herbs were found, only one of which was also included in the ‘Mix-Herb’ seed mixture. *Holcus lanatus* was the only grass species that was introduced from outside into both vegetation areas and ultimately accounted for a considerable proportion of 5% of the ‘Mix-Herb’ and 2% of the ‘Mix-Grass’ plant community. For all other newly introduced plant species, the proportion in the respective plant communities was less than 3% (see S1 Table).

### 3.3 Drought periods received from SPI values

**Fig 3:**
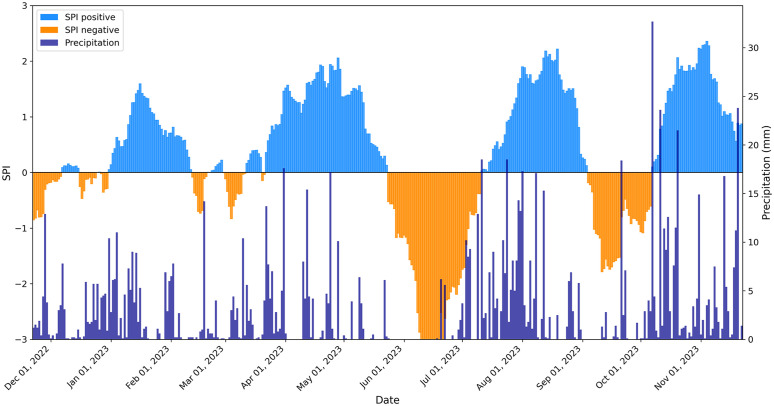
Detecting meteorological drought periods based on SPI values and precipitation history. Standard Precipitation Index (SPI) based on precipitation reference data from 1991 to 2020 from the weather station Burhave from November 22, 2022 to November 22, 2023 and corresponding daily precipitation. Periods, which by definition represent a drought, correspond to the negative SPI values (orange), whereby a distinction is made between moderate (−1.00 to −1.49 SPI), severe (−1.50 to −1.99 SPI) and extreme events (≤−2.00 SPI; [56]).

In the observed time span from November 2022 to November 2023, the daily SPI values with a rolling 30-day average revealed large variations between wet and dry periods. Particularly striking was a dry period that lasted from May 24, 2023 to July 10, 2023, in which negative SPI values down to a minimum value of −3 were documented (Fig 3). This drought period was initiated by a total of 24 precipitation-free days, which lasted from the end of May to mid-June. The minimum SPI value of −3 from June 10, 2023 to June 19, 2023 correspond to an extreme drought period with a probability of occurrence of 2.3% [56].

Another drought period was documented for the second half of the year, starting on September 4, 2023 and lasting until October 6, 2023. On September 11, 2023, SPI values dropped down to −1.79, indicating a severe drought with a probability of occurrence of 4.4% (Fig 3; [56] McKee et al. 1993). In order to be able to better compare both drought periods in 2023, the following discussion primarily focused on the days from the first negative SPI value until similar SPI values of −1.75 in June 6, and −1.79 in September 11, 2023 were documented.

Three of the four heaviest daily precipitation occurred directly after the second drought period, namely on October 7, 2023 with 32.7 mm m^-2^ and on October 11 and 20, 2023, with 23.6 mm m^-2^ and 21.5 mm m^-2^ respectively. This phase of heavy precipitation resulted in the wettest phase with highest SPI values in the study period with a maximum of 2.4 on November 4, 2023 (Fig 3). On November 20, 2023 23.8 mm m^-2^ of precipitation were recorded.

## 4. Discussion

### 4.1 Differences in plant functional traits and their role in thermal soil stability

**Fig 4:**
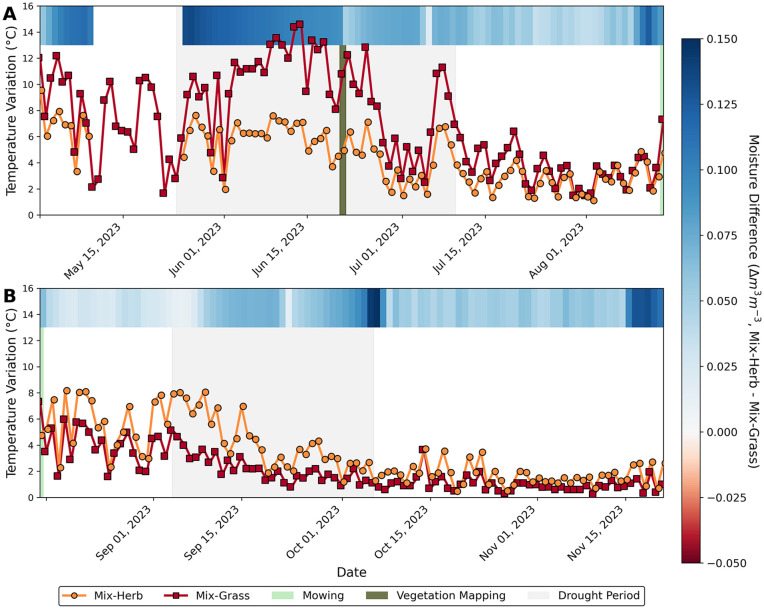
Changes in soil physical conditions as a function of mowing. Diurnal temperature variations (difference between daily maximum and minimum soil temperature) of ‘Mix-Herb’ (orange circles) and ‘Mix-Grass’ (red squares) recorded at a depth of 4 cm for the periods May 1, 2023 to August 13, 2023 (before mowing; A), and August 15, 2023 to November 22, 2023 (after mowing; B). Heatmap strip represents daily average differences in soil moisture between the ‘Mix-Herb’ and the ‘Mix-Grass’ vegetation areas. Grey shaded bands highlight both drought periods (negative SPI values), while olive green and light green bands mark the vegetation mapping and mowing event, respectively. Data gap between May 9 and May 25, 2023 on the ‘Mix-Herb’ dike section is due to a temporal sensor breakdown.

In the face of rising air and soil temperatures and longer periods of drought due to climate change, vegetation communities that better retain soil moisture and reduce soil temperature variations appear to be a suitable measure for stabilizing the microclimate. The measurements of the soil temperatures during the first drought period showed that not only the diurnal temperature variations on both dike sections studied were different, namely higher variations on the ‘Mix-Grass’ area, but also the general average soil temperature varied greatly depending on the vegetation area, namely lower average temperatures on the ‘Mix-Herb’ area (Figs 2 and 4). Similar patterns were also observed in other studies. Higher plant diversity – including species-rich herb mixtures – led to greater stabilization of soil temperature, with both diurnal averages and minimum-maximum variations being considerably reduced [59]. The authors attribute their observations to a denser vegetation structure, increased shading and an improved soil structure with a higher organic carbon content, which reduces heat conduction in the soil. Different compositions of grass species (Poaceae) and legume species (Fabaceae) of contrasting diversity were also tested and found that higher plant diversity leads to greater temporal stability of plant production in the ecosystem, especially in years with extreme climatic conditions [15]. This supports the biodiversity-stability hypothesis [29] and suggest that diverse plant communities, such as those established on the ‘Mix-Herb’ dike section, can apparently better buffer and stabilize soil microclimate conditions under variable environmental regimes through their higher species richness and variation in functional and structural traits.

However, after the mowing was carried out on both dike sections in Butjadingen on August 14, 2023, the previously observed effect of lower diurnal temperature variations in the soil of the herb-dominated plant community was reversed (Fig 4B). During the drought period from September 04, 2023 to September 11, 2023 the diurnal variations in soil temperature in the ‘Mix-Herb’ vegetation area were now becoming higher (7.2 °C on average), even though soil moisture was still higher than in the ‘Mix-Grass’ vegetation area (Figs 2 and 4). The diurnal temperature variations in the soil of the ‘Mix-Grass’ area were noticeably lower in the same period, averaging 3.7 °C. The reversed trend of the diurnal variations in soil temperature remained until the end of this study on November 22, 2023 (100 days after mowing). This observation suggests that the lower diurnal temperature variations in the soil are not solely attributable to the high heat storage capacity caused by a higher soil moisture [60,61], but rather indicating a substantial influence of above-ground biomass. Before mowing, on June 21, 2023, the general height of the plants on the ‘Mix-Herb’ vegetation area was 50–150 cm, which was notably higher than the plant height on the ‘Mix-Grass’ area that was only around 60 cm. It can be assumed that the broader range of functional traits of the herb-dominated plant community prior to mowing likely contributed to greater shading [59], improved evaporative cooling and radiation balance as well as reduced wind circulation, resulting in smaller temperature variations in the soil. These functional traits, as given by a plant community with a high number of herbaceous species, appear to have had a positive effect on the microclimate in the topsoil, which could be observed particularly during the first drought period. In contrast, the higher soil temperatures in the ‘Mix-Grass’ area can possibly be attributed to non- or loosely tufted grasses, as is characteristic of most *Festuca* species and *Phleum pratense*, resulting in a rather low soil shading and a low transpiration rate during the first drought period [62]. Interestingly, however, cut plant residues are also considered to have an influence on the soil properties and it is generally recommended to refrain from residual removal, as unmulched soils are otherwise subject to accelerated evaporation, elevated diurnal variations in soil temperature and soil erosion [63]. In the present study, however, differentiated effects were observed after mowing: As stated above, the diurnal variations in soil temperature on the previously more favorable, herb-rich vegetation area now increased considerably after mowing, while the temperature variations on the grass-dominated area were comparatively lower – despite the lower soil moisture there (Fig 4B). One explanation for the different occurrence of temperature variations after mowing could be differences in plant density. After mowing, high plant density can still shade the ground better despite the short vegetation length than low plant density, where the bare ground is exposed. Plant density is linked to biodiversity, with plant communities with grasses having a higher plant density than those without grasses [64]. The higher percentage of 46% grasses in the ‘Mix-Grass’ seed mixture compared to 40% grasses in the ‘Mix-Herb’ area during vegetation mapping in June 2023 may have led to a higher plant density in the ‘Mix-Grass’ area. However, the herbs, which grow to a height of 50–150 cm, are considerably taller than the grasses, shading the ground and displacing low-growing plants, resulting in a lower plant density [64,65], which can then lead to higher temperature variations after mowing. Another explanation could be due to structural differences in the mulch material: The looser litter of herbaceous species may have provided less effective protection against direct radiation and nighttime cooling, while the denser layer of grass mulch seemed to have a stronger isolating effect. In addition, the residues from herbaceous plants decompose much faster due to their higher nitrogen and lower lignin content, resulting in a rapid loss of the protective effect of a mulch layer [66]. In contrast, the structurally more persistent grass residues can remain on the soil surface for much longer, partly due to silicon accumulation in their cell walls, providing more sustained protection against temperature extremes [67,68]. This means that dike maintenance measures, in particular the timing of mowing but also the management of cut plant residues, can have a decisive influence on the microclimatic and local environmental conditions for the dike vegetation.

Since summer mowing appears to increase the risk of soil heating, mowing should be avoided during periods with the greatest probability of drought occurrence. However, shifting mowing to spring could impair flowering and seed production, whereas mowing in early-summer (June) usually allows regrowth and re-flowering in late-summer [69], but this could then coincide with periods of drought. In contrast, postponing mowing to winter times could increase the risk of soil erosion due to storm surges, as wave dissipation is lower on mowed surfaces [70]. Since mowing ultimately mimics grazing pressure, similar findings from sustainable grazing management could be considered in mowing strategies [71]. Accordingly, spatially mosaic mowing strategies and/or heterogenous mowing times could help maintain functional plant diversity without suppressing flowering [69] or risking the loss of a protective vegetation coverage. Where feasible, cut plant residues should ideally remain on the mowed areas, at least during a drought period, to avoid accelerated soil heating and dehydration.

### 4.2 Variability of soil moisture as a result of plant species composition

The vegetation mapping carried out in June 2023 suggests that depending on the prevailing plant communities, different root systems had developed in the two dike sections studied, allowing water resources to be accessed and retained in the soil in different ways (e.g., [72–74]). After all, a higher annual soil moisture by 0.054 m^3^ m^-3^ ± 0.018 (75% percentile) was found for the ‘Mix-Herb’ dike section, which was dominated by herbaceous plants, compared to the soil covered with mainly grass species (‘Mix-Grass’). These contrasting results of the soil moisture measurements on both dike sections are consistent with earlier studies, which showed that grasses primarily draw water from the upper soil layers and thus can also deplete it more quickly. In particular, short grasses usually lack a deep and dense root system [75–77], but typically have a fibrous root system characterized by a dense network of fine roots, concentrated primarily in the upper soil layers [78], reaching depths of up to 1 m [79]. These roots enable the grasses to efficiently capture water from light rainfall events, which often only wet the surface soil [78].

**Fig 5:**
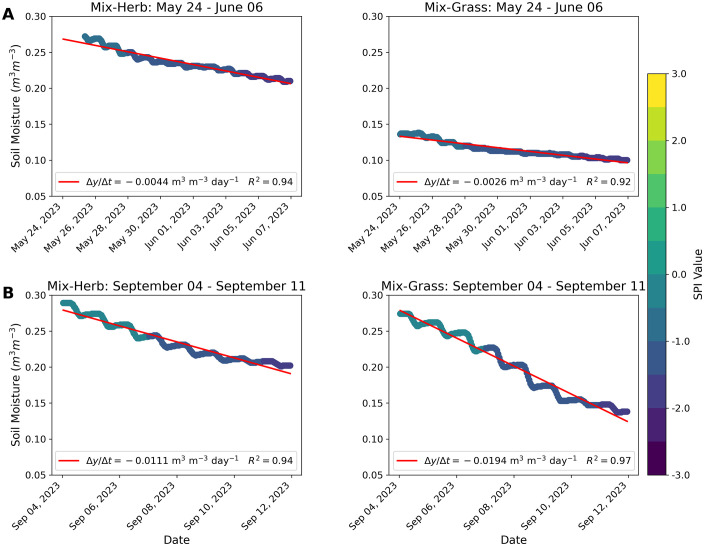
Influence of vegetation composition on the rate of soil moisture loss during drought periods. (A) Soil moisture during the first phase of the drought period from May 24, 2023 to June 6, 2023 with SPI values from −0.53 to −1.75 and linear regression with soil moisture change (Δy) over time (Δt) in days. (B) Soil moisture during the first phase of the drought period from September 04, 2023 to September 11, 2023 with SPI values from −0.19 to −1.79 and linear regression with soil moisture change (Δy) over time (Δt) in days.

The contrasting influence of both different plant communities on soil moisture was particularly evident in the first phase of the drought period in summer 2023 (from May 24, 2023 to June 6, 2023 [see Chapter 3.3]), when soil moisture in the herb-dominated vegetation area decreased almost twice as quickly as in the grass-dominated area. There, the loss rates of soil moisture reached 0.004 m^3^ m^-3^ d^-1^ for the ‘Mix-Herb’ area (R² = 0.94), compared to 0.003 m^3^ m^-3^ d^-1^ for the ‘Mix-Grass’ area (R² = 0.92; Fig 5A). Moisture loss through evaporation is higher in wetter soils, as it was at the beginning of the drought period on May 25, 2023 at the ‘Mix-Herb’ vegetation area with 0.268 m^3^ m^-3^ compared to 0.133 m^3^ m^-3^ at the same time at the ‘Mix-Grass’ vegetation area, because they contain more liquid water that is freely available in their pores [80]. In moister soils, water is less tightly bound allowing liquid water to rise more easily to the soil surface through connected pores and capillary potential ([81] and references therein). This promotes a higher evaporation rate, as the soil can continuously supply water to replace the evaporated water [82]. As the soil dries out, the water potential becomes more negative, the water becomes more tightly bound to the soil particles and the transport of water to the surface slows down, reducing evaporation [82]. Despite the higher moisture decrease in the herb-dominated soil, the absolute soil moisture remained on average 0.098 m^3^ m^-3^ higher than in the soil of the grass-dominated area during the period of maximum drought from June 10, 2023 to June 19, 2023, when the SPI fell to −3. These different patterns in soil moisture loss but also in moisture retention during a drought may be attributable to the fact that most herb species (Asteraceae, Fabaceae etc.) exhibit a greater variety in root architecture, often including taproots that can penetrate deeper into the soil profile, reaching depths of 2–4 m, alongside a network of finer, shallower roots [79,83,84]. This dual-layered and more complex root system allows the herbs to access water resources at varying depths within the soil more efficiently [83] and to already adsorb soil water from below the root zone of grasses [76,79]. While the shallow, dense roots of grasses are advantageous for capturing surface moisture, their access to deeper water reserves may be limited, especially during prolonged dry periods when the upper soil layers are depleted [85]. For a grass-dominated dike vegetation, such as the ‘Mix-Grass’ area, this means that moderate precipitation conditions may be sufficient to maintain a healthy vegetation coverage, while during dry periods they appear to reach their functional limit, which may be reflected in a decline in near-surface soil moisture. In addition, although herbs often grow in association with grasses, as was also mimicked on the ‘Mix-Herb’ dike section, they grow faster than the grasses and thus often overgrow them, resulting in little competition from grasses [79].

From September 04, 2023 to September 11, 2023 SPI values again dropped noticeably and indicated another drought period (see Chapter 3.3 and Fig 3), with two different initial conditions compared to the one from May to June: The initial soil moisture difference between both areas was very small, at 0.014 m^3^ m^-3^ and the dike sections were mowed prior to the second drought on August 14, 2023. The soil moisture loss rates were this time much higher on the grass-dominated vegetation area (‘Mix-Grass’: 0.019 m^3^ m^-3^ d^-1^, with R² = 0.97) compared to the herb-dominated area (‘Mix-Herb’: 0.011 m^3^ m^-3^ d^-1^, with R² = 0.94). As the soil moisture in the soil of the ‘Mix-Herb’ area was nearly the same at the beginning of both drought periods, the difference in the soil moisture loss rate can be attributed to the shortage of above-ground biomass and thus higher soil temperature due to a lack of shade and higher evaporation due to the missing windbreak and higher soil temperature. The loss rate of soil moisture was 2.5 times higher in September 2023 and after mowing. In contrast, the soil of the ‘Mix-Grass’ area in September 2023 was almost as moist as that of the ‘Mix-Herb’ area, and thus twice as moist as at the beginning of the first drought period in May 2023. With the moister soil in September 2023 and the mowing took place the soil moisture loss rate of the ‘Mix-Grass’ area was 7.5 times higher compared to May. Apparently, the initial soil moisture seems to have a high impact on the moisture loss rate during a drought. Besides this, not only the below-ground plant traits, but also the removed above-ground biomass in combination with the mowed residues remaining on the area seemed to have an influence on the soil moisture loss rate.

Since the soil moisture difference between the two vegetation areas during the drought in May to June 2023 was even higher than the average yearly difference, it can be assumed that the herb-dominated plant community with its more diverse functional traits – including deep-reaching root systems – was able to access deeper water resources more efficiently and possibly also keep near-surface soil layers moister through hydraulic lift ([74,86] and references therein). Interestingly, however, these patterns of different dike section-related soil moisture and functional classifications are not completely reflected in the respective species composition: For example, deep-rooting ‘herb’ species such as *A. millefolium* (Asteraceae) and *L. corniculatus* (Fabaceae) were also abundant in the ‘Mix-Grass’ plant community dominated by grasses. In contrast, grasses are also abundant on the ‘Mix-Herb’ area dominated by herbaceous species – including *A. elatius* (Poaceae). However, this perennial species is a deep-rooted grass whose root traits are likely to be similar to those of the surrounding herbaceous species [87]. Moreover, if precipitation is low or absent for a long time, root systems tend to become shallower overall. This can be particularly problematic for shallow-rooted grasses, as competition for water resources in the upper soil layers intensifies [76]. Herbs, by contrast, with their capacity to extend roots to greater depths, can tap into water sources that are still available even when the surface soil has already dried out. Furthermore, a positive feedback between plant diversity and soil moisture can be assumed, with both effects likely to reinforce each other. Previous studies have shown that communities with greater species richness and functional trait diversity retain soil moisture more effectively during periods of drought, while at the same time, favorable soil moisture conditions may support the maintenance of higher diversity [88]. This highlights the intertwined role of biodiversity and hydrology in stabilizing soil microclimate and ecosystem functioning.

### 4.3 The impact of plant biodiversity on the resistance of dike soils

The two dike sections studied – with the ‘Mix-Herb’ and ‘Mix-Grass’ plant communities – differed not only in terms of their morphological and physiological vegetation structure, but also in terms of the prevailing species diversity at the taxonomic level. Looking at the whole plant composition, it was found that although both Shannon Indices decreased after seeding, the Shannon Index remained higher on the ‘Mix-Herb’ vegetation area (decline from 2.7 to 2.3) than on the ‘Mix-Grass’ area (decline from 2.2 to 2.0). Hence, the difference in the Shannon Index between the two areas studied was rather small and thus indicated moderate to slightly elevated diversities for both areas in June 2023. Species richness, on the other hand, had increased considerably on both vegetation areas since sowing, although the values remained higher on the ‘Mix-Herb’ area (herb species: stayed 15; grass species: increase from 1 to 12) than on the ‘Mix-Grass’ area (herb species: increase from 5 to 10; grass species: increase from 5 to 8). The reason for this may be that the Shannon Index and species richness are two different variables that are not fully comparable [89], as the Shannon Index considers not only the number of species, but also the uniformity (evenness) with which species occur in abundance [90]. It demonstrates that on the ‘Mix-Herb’ area, the plant species are more evenly in number, resulting in a higher Shannon Index. In contrast, on the ‘Mix-Grass’ area, individual plant species dominate strongly, while most other species are rather rare, resulting in a lower Shannon Index.

Since the Jaccard index was used to calculate β-diversity and compare the similarity of the seed mixtures and established plant communities, it was found that 24% of the total species were identical at the time of sowing. At the time of vegetation mapping, however, this was 29% of the total species, which means that the plant communities became somewhat more similar over a period of two years. Natural succession of plant communities is an inherent process, especially in experimental setups where adjacent vegetation areas are established under identical environmental conditions to ensure comparability. An exchange of species between them is therefore almost inevitable. At the time of vegetation mapping, however, the proportion of species originating from the respective other seed mixture was very low and thus had no relevant impact on the overall results. Since research on dike vegetation with varying diversity remains scarce, comparable results are only available to a limited extent. A 7% drop in β-diversity over two years was found in grassland systems [91], which is about the same order of magnitude as in the present study. However, because the sites investigated were grazed [91], this likely produced the opposite pattern and does not explain the fact that the β-diversity increased in this study.

In principle and according to the biodiversity-stability hypothesis, species-rich plant communities help systems to respond better to environmental changes and buffer extreme climatic events through complementary use of resources, increased productivity and stabilization of ecosystem processes [29,92–94]. The underlying reason why, despite only marginal differences in the Shannon Index, the plant community of the ‘Mix-Herb’ area showed considerably more positive effects on the soil temperature and moisture than that of the ‘Mix-Grass’ area – especially during prolonged drought periods – may be the contribution of individual plant species. In fact, the morphological as well as physiological traits of the corresponding species, but also the more diverse composition of the ‘Mix-Herb’ plant community (both in terms of taxonomy and morphology) could have favored greater shading, reduced soil temperature and reduced evaporation and thus maintained soil moisture. In particular, the species that cover the soil over large areas with stolons (e.g., *L. corniculatus* and *Potentilla anserina*), have deep roots or rhizomes (e.g., *C. intybus* and *P. lanceolata*), or have a high shading effect due to their leaf arrangement and shape (e.g., *Artemisia maritima* and *Trifolium* spp.), seem to contribute particularly to better moisture conditions [95]. Although grasses can also have deep and dense root systems and could therefore positively influence soil moisture as well, plants with broader leaves and/or far-reaching stolons and rhizomes may contribute much better to soil moisture conservation, as these plant structures can protect the soil from wind and reduce evaporation, further minimizing soil drying out [96].

The sole number of species, or biodiversity in the taxonomic sense, is therefore not the only decisive factor for the stability of a system. Instead, as this study showed, functional heterogeneity within a plant community also plays a key role in resistance to environmental stress, as different species occupy different ecological niches [97]. Even though the ‘Mix-Herb’ area had a similarly high Shannon Index as the ‘Mix-Grass’ area, the plant community of the latter still tended to have a low functional diversity, as the grass species involved seemed to be ecologically too similar. Accordingly, this vegetation area was more susceptible to drought periods and lack of precipitation than the more functional heterogenous ‘Mix-Herb’ area. Overall, this functional heterogeneity (or diversity) could have led to an ecological complementarity in which the different herbaceous plants complemented each other in their services and thus ensured the stability of the dike soil during the study period [98].

### 4.4 Limitations

This study provides valuable insights into the influence of plant traits of plant communities with different species diversity on soil temperature and moisture on a dike, but several limitations must be considered when interpreting the results.

First of all, from May 9 to May 25, 2023, one of the soil sensors failed, meaning that the first day of the drought period could not be recorded by the soil sensor on the ‘Mix-Herb’ area. This failure resulted in a temporary loss of data continuity. To mitigate potential data gaps in future comparable studies, it would be advisable to install multiple soil sensors at the same depth.

Furthermore, a primary constraint was the reliance on a single seeding event at the study area, meaning that the subsequent plant community development was intrinsically linked to the environmental conditions prevailing at that specific time. In addition, natural succession could not be avoided in an only partially controlled setting in the field, presumably leading to assimilation of the plant communities over time, but also to foreign species being sown naturally and establishing themselves in the vegetation areas. For future investigations, it would be advantageous to conduct studies at sites with more stable and fully developed plant communities or to setup the experiment at spatially independent locations, while ensuring comparable climatic and environmental conditions. This would have allowed for a more robust assessment of different seeding mixtures under established ecological conditions without interference. However, such a site selection and separation was not feasible in the present study due to permit constraints imposed by the coastal authority.

Another important consideration for future experimental setups is the differential contribution of various plant functional traits to the observed soil temperature and moisture dynamics. For instance, while plants with taproots can enhance soil water storage capacity, the introduction of these plants on the dike is often viewed critically from a coastal protection perspective. Taproots generally contribute less to the structural integrity and erosion resistance of coastal defense structures compared to fibrous, shallow root systems. Therefore, a more detailed analysis of the specific plant species contributing to the observed soil physical benefits is warranted.

With regard to mowing, the consideration of only one mowing regime may represent a limitation of this study. Future research should include different maintenance regimes and timings to better capture their potentially varying effects on vegetation and soil conditions. In order to avoid over-exploitation of the vegetation areas through intensive management and in line with the system that had existed up to that point, mowing was only carried out once per year, so that only one mowing event took place during the period covered by the soil sensors. In order to be able to make more reliable statements about how a plant community recovers after mowing, i.e., how resilient the individual vegetation areas are, such interventions would also have to be reproduced.

The Standardized Precipitation Index (SPI) was used to classify drought periods, as it represents a robust and widely applied metric for assessing precipitation deficits. While the Standardized Precipitation Evapotranspiration Index (SPEI; e.g., [99]) provides an extended measure of climatic water deficits by incorporating potential evapotranspiration, it was not applied in this study because the required air temperature data were only available from a weather station located 18 km away (Bremerhaven), which would not adequately reflect local site conditions. For future studies where local air temperature data are available, however, the SPEI could serve as a valuable complement for a more detailed assessment of drought conditions.

Finally, this study did not delve into the broader implications for coastal defense, an aspect that should be a central focus of future research. Investigating the interplay between plant communities and the structural integrity of coastal protection measures is crucial for informed management strategies.

## 5. Conclusions

The promotion of biodiverse dike vegetation could be a small contribution, but a practical application in the implementation of ecological goals, such as reversing the decline in biodiversity in Germany and turning it into a positive trend, as the UN sustainability goals of the federal ministry for the environment, climate protection, nature conservation and nuclear safety (BMUKN) are aiming for. Dikes are and must be designed primarily for flood protection [100], but could also act as ecological corridors and connect fragmented habitats.

Hence, the aim of this study was to investigate the influences of plant species richness and functional trait diversity on soil temperature and moisture and thus the climate resistance and sustainable functionality of dike vegetation. These investigations revealed significant differences in soil temperature variations and average soil moisture for the plant species compositions tested on the different dike sections. During the study period, the herb-dominated seed mixture (‘Mix-Herb’) showed higher soil moisture and lower soil temperature as well as lower diurnal temperature variations before mowing compared to the grass-dominated seed mixture (‘Mix-Grass’). It was also observed that the soil moisture on the ‘Mix Herb’ vegetation area remained similar and higher after mowing, while the diurnal soil temperature variations increased compared to ‘Mix-Grass’. Most likely, the herbaceous species, especially those belonging to the Asteraceae, Fabaceae and Plantaginaceae, their deeper and structurally more diverse root systems, enabled improved access to deeper soil water reserves on the dike section studied and thus supported the maintenance of soil moisture – even after mowing and under drought conditions. This is a great advantage during prolonged droughts and in times of high evaporation rates, and should be considered when deciding on dike vegetation.

The microclimate created by the different plant traits in turn seems to have influenced the soil temperature and the evaporation rates. The initially higher diurnal temperature variation in the soil of the ‘Mix-Grass’ area, potentially linked to the drier soil and less shadowing and its reversal after mowing, with higher temperature variations in the soil of the ‘Mix-Herb’ area, highlight the complex interplay between vegetation structure, soil moisture and microclimate. While above-ground biomass may influence temperature variation, root system architecture and greater biodiversity appear to be the driving mechanisms for water storage capacity and soil moisture retention. In this context, the diversity of plants – for example in the form of grasses and herbs – is particularly important, as they have different eco-physiological strategies and root architectures. These findings underscore the ecological importance of (functional) heterogenous plant communities in influencing soil hydrological and thermal properties, particularly in the context of drought resistance under ongoing climate change.

In order to ensure optimal conditions for soil and vegetation properties and thus, in the best case, to strengthen their inherent resistance to more extreme climatic impacts, mowing and grazing could in the future be timed in such a way that the dike vegetation retains soil moisture longer through plant-based shading and density in order to avoid desiccation cracks before expected drought periods. Implementing spatially and temporally heterogeneous mowing schedules adapted to seasonal stress periods may help to balance vegetation resistance, thereby mitigating soil heating during drought periods while conserving functional diversity.

## Supporting information

S1 Table Overview of sown and subsequently established plants. Plant species with the respective number of seeds per square meter sown on the dike sections ‘Mix-Herb’ and ‘Mix-Grass’ in September 24, 2021 and the relative plant species abundancy (by degree coverage) mapped in June 21, 2023 on the respective dike sections.
(DOCX)

S1 Fig Temporal change in soil temperature and moisture across different soil depths. Hourly average soil temperature (light and dark red line) and soil moisture (light and dark blue line) during the measurement period from November 22, 2022 to November 22, 2023, comparing the ‘Mix-Herb’ (light) and ‘Mix-Grass’ (dark) dike sections at depths of 4 cm (A), 14 cm (B) and 24 cm (C). Data gaps between May 9 and May 25, 2023, on the ‘Mix-Herb’ dike section at a depth of 4 cm and on the ‘Mix-Grass’ section at a depth of 24 cm as well as from August 19, 2023 at a depth of 14 cm on the ‘Mix-Grass’ section were due to temporary sensor breakdowns.
(TIF)
