# Supplementary material for: Plant trait diversity buffers soil moisture dynamics on coastal dikes during drought periods
Source: PLoS One. 2026 Mar 26;21(3):e0345552. doi: 10.1371/journal.pone.0345552 (PMC13020843; doi:10.1371/journal.pone.0345552)
Supplement: S1 Table — Plant species with the respective number of seeds per square meter sown on the dike sections ‘Mix-Herb’ and ‘Mix-Grass’ in September 24, 2021 and the relative plant species abundancy (by degree coverage) mapped in June 21, 2023 on the respective dike sections. (DOCX) [file pone.0345552.s001.docx]

|  | **‚Mix-Grass‘** | | **‚Mix-Herb‘** | |
| --- | --- | --- | --- | --- |
| Plant species | Sowing on September 24, 2021 [seeds m^-^²] | Mapping on June 21, 2023 [%] | Sowing on September 24, 2021 [seeds m^-^²] | Mapping on June 21, 2023 [%] |
| Achillea millefolium | 359.00 | 14.00 | 360.00 | 1.83 |
| Centaurea jacea |  |  | 838.00 | 3.00 |
| Cichorium intybus |  | 0.17 | 601.00 | 15.00 |
| Cirsium arvense |  |  |  | 0.50 |
| Knautia arvensis |  |  | 601.00 | 2.50 |
| Leucanthemum vulgare |  |  | 846.00 | 22.50 |
| Lotus corniculatus | 356.00 | 22.50 | 599.00 | 6.50 |
| Malva sylvestris |  |  | 601.00 | 2.50 |
| Medicago lupulina |  |  | 841.00 | 0.33 |
| Pastinaca sativa |  |  |  | 0.33 |
| Plantago lanceolata | 356.00 | 15.00 | 840.00 | 2.00 |
| Prunella vulgaris |  |  | 718.00 | 1.00 |
| Rannuculus repens |  | 0.20 |  |  |
| Rorippa sylvestris |  | 0.20 |  |  |
| Rumex crispus |  | 0.17 |  |  |
| Scorzoneroides autumnalis |  |  | 599.00 |  |
| Sonchus asper |  | 0.17 |  |  |
| Sonchus oleraceus |  | 0.17 |  |  |
| Trifolium dubium |  |  | 602.00 | 0.40 |
| Trifolium fragiferum | 473.00 |  | 1080.00 |  |
| Trifolium pratense |  |  |  | 0.73 |
| Trifolium repens | 475.00 | 1.17 | 876.00 |  |
| Viccia cracca |  |  | 360.00 | 0.73 |
| Herbs | **∑ 2,019.00** | **∑ 53.73** | **∑ 10,362.00** | **∑ 59.87** |
| Agrostis capillaris | 1235.00 | 0.70 |  | 2.83 |
| Alopecurus pratensis |  |  |  | 0.40 |
| Arrhenatherum elatius |  |  | 227.00 | 22.50 |
| Bromus hordeaceus |  |  |  | 0.33 |
| Cynosurus cristatus | 881.00 | 0.37 |  | 0.50 |
| Elymus farctus |  |  |  | 2.50 |
| Elymus repens |  |  |  | 0.33 |
| Festuca pratensis | 905.00 | 17.50 |  | 0.50 |
| Festuca commutata |  | 0.20 |  |  |
| Festuca rubra | 2558 | 12.50 |  | 1.50 |
| Holcus lanatus |  | 2.00 |  | 5.00 |
| Phleum pratense | 2432 | 12.50 |  | 2.90 |
| Poa pratensis |  | 0.50 |  |  |
| Poa trivialis |  |  |  | 0.83 |
| Grasses | **∑ 8,012.00** | **∑ 46.27** | **∑ 227.00** | **∑ 40.13** |
