# Supplementary figures and images for: Plant trait diversity buffers soil moisture dynamics on coastal dikes during drought periods
Source: PLoS One. 2026 Mar 26;21(3):e0345552. doi: 10.1371/journal.pone.0345552 (PMC13020843; doi:10.1371/journal.pone.0345552)

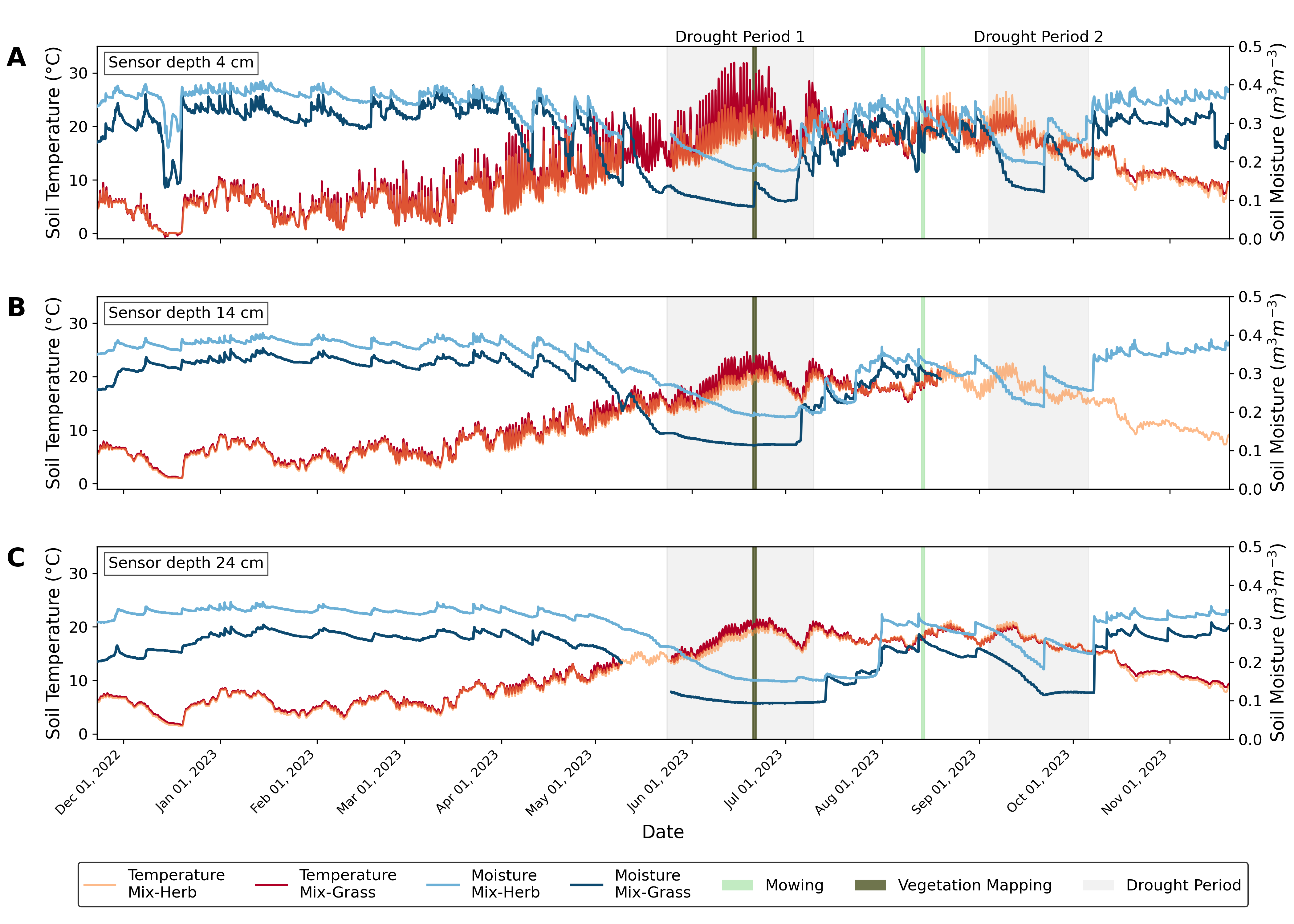

Supplement: S1 Fig — Hourly average soil temperature (light and dark red line) and soil moisture (light and dark blue line) during the measurement period from November 22, 2022 to November 22, 2023, comparing the ‘Mix-Herb’ (light) and ‘Mix-Grass’ (dark) dike sections at depths of 4 cm (A), 14 cm (B) and 24 cm (C). Data gaps between May 9 and May 25, 2023, on the ‘Mix-Herb’ dike section at a depth of 4 cm and on the ‘Mix-Grass’ section at a depth of 24 cm as well as from August 19, 2023 at a depth of 14 cm on the ‘Mix-Grass’ section were due to temporary sensor breakdowns. (TIF) [file pone.0345552.s002.tif]
